# Introducing the Novel Deceleration Threshold Method: Comparative Reliability to Previous Sprint Deceleration Analysis Methods in Team‐Sport Athletes

**DOI:** 10.1002/ejsc.12278

**Published:** 2025-03-05

**Authors:** Millicent A. West, Heidi R. Compton, Ben J. Dascombe, Josh L. Secomb

**Affiliations:** ^1^ Applied Sport Science and Exercise Testing Lab School of Biomedical Sciences and Pharmacy University of Newcastle Ourimbah Australia; ^2^ School of Health Sciences Western Sydney University Campbelltown Australia; ^3^ Active Living and Learning Research Program Hunter Medical Research Institute Callaghan Australia

**Keywords:** coefficient of variation, inter‐day, intra‐day, intraclass coefficient, pre‐planned

## Abstract

Pre‐planned deceleration assessments are vital for profiling team sport athletes as they provide a measure of the athletes' ability to implement effective performance and protection strategies to tolerate the substantial mechanical forces. Although task requirements of these linear assessments are generally consistent, current research employs low sampling technology, which limits the ability to define the deceleration start point and subsequently the reliability. Therefore, this study compared the intra‐ and inter‐day reliability of three deceleration assessment analysis methods: set distance, peak velocity, and the novel deceleration threshold, using a motorized linear encoder device. Ten female and 10 male subjects performed three maximal effort 30 m sprint deceleration trials over two testing days. Each trial was filtered and analyzed using a customized code to calculate key deceleration metrics: distance‐to‐stop (DTS), time‐to‐stop (TTS), and average deceleration (DEC_ave_) for each analysis method. Intraclass correlation coefficients (ICC) using two‐way fixed effect models, coefficients of variation (CV%), and standard errors of measurement (SEM) were calculated to determine the intra‐day reliability across all three trials of the first testing day, and inter‐day reliability using the: best, average of best two, and average of all trials. The deceleration threshold method using the average of the best two trials from each testing day, exhibited excellent inter‐day reliability for the key metrics (TTS: ICC = 0.93, CV% = 6.4; DTS: ICC = 0.97, CV% = 5.3; DEC_ave_: ICC = 0.92, CV% = 7.1). To optimize reliability and sensitivity of detecting a meaningful change in sprint deceleration performance practitioners should consider using the average of two best trials analyzed with the deceleration threshold.

**Summary**

- Current methods for linear deceleration assessments use equipment with relatively low sampling equipment, affecting the precision of determining the deceleration start point and thus reliability. Therefore, using higher sampling rate equipment and a deceleration threshold may enhance sensitivity and improve reliability of these assessments.
- The acceleration data collected in this study indicates that most subjects began decelerating prior to the 30 m mark and did not consistently initiate deceleration immediately after reaching peak velocity, likely contributing to the lower reliability for the set distance and peak velocity methods.
- The deceleration threshold method demonstrated superior intra‐day reliability for distance‐to‐stop and inter‐day reliability for all key deceleration metrics when using the average of the best two trials for analysis.
- This study provides practitioners with an alternate deceleration analysis method that may enhance reliability and the likelihood of detecting meaningful changes in performance.

## 1 Introduction

Deceleration efforts are important locomotive movements in a variety of team‐sports that are performed as frequently as accelerations, to reduce momentum after a sprint effort (McBurnie et al. 2022). Such efforts are crucial for competitive performance, as a faster and more efficient deceleration can enhance success when avoiding or pursuing an opponent in both offensive and defensive situations, respectively (D. J. Harper, Jordan, and Kiely 2021; Martínez‐Hernández, Quinn, and Jones 2023). As such, assessments of pre‐planned linear decelerations are becoming increasingly utilized in team‐sport environments as the demand to develop practitioners' knowledge of an athlete's capability in these tasks are warranted given the relationship to performance and injury risk (D. J. Harper and Kiely 2018; McBurnie et al. 2022). The advantages of linear deceleration assessments are the ability to profile the velocity, distance, and duration variables of performance, which can be used to guide individualized technical and physical training programs (D. Harper et al. 2024). As such, it is crucial that practitioners have access to assessment methods that are both valid and reliable in determining an athlete's maximal deceleration capability to ensure effective benchmarking and monitoring of performance.

Current linear deceleration assessment protocols require the athlete to perform a maximal effort sprint to a specified set distance; prior to decelerating to a stationary stop as rapidly as possible. To quantify the deceleration performance, the start point of the deceleration must be set, with current methods either setting this point from the end of the set distance or immediately after the point of peak velocity (Ashton and Jones 2019; D. J. Harper et al. 2023). However, in contrast to reactive assessments, pre‐planned deceleration efforts allow for preparatory adjustments to body position and approach velocity, which may assist in protecting the body from the substantial mechanical forces experienced during execution (McBurnie et al. 2022; Young et al. 2022). Therefore, it may be that the cognitive component of preparing for the rapid deceleration could result in a conscious or unconscious initiation of this effort prior to the specified set distance. This may result in variance in the point that the deceleration effort is initiated.

The most commonly reported pre‐planned deceleration assessment is the acceleration–deceleration ability (ADA) test, which requires the athlete to sprint for a set distance of 20 m before initiating a maximal effort deceleration (D. J. Harper, Jordan, and Kiely 2021). Performance is typically measured using a combination of radar technology and timing gates, which allow for simple computational analysis of the key deceleration metrics, including: distance‐to‐stop (DTS), time‐to‐stop (TTS), and the average of all instantaneous deceleration (negative acceleration) values across the deceleration effort (DEC_ave_) (D. J. Harper et al. 2023). For the ADA assessment, these key deceleration metrics are analyzed by setting the start point as the end of the sprint distance (i.e., 20 m; set distance method). However, in a follow‐up study D. J. Harper et al. (2023) determined that the mean distance where the peak velocity was achieved during the 20 m sprint effort was at a position of 17.2 m. Therefore, as the subjects were determined to be decelerating approximately 2.8 m prior to the 20 m set distance, it was suggested to use the point where the peak velocity occurred as the start of the deceleration effort (peak velocity method). However, this method may also be vulnerable to reduced reliability if there is any measurement error, as can be observed in equipment with a relatively low sampling frequency, such as radar, or if the athlete achieves an instantaneous peak velocity that cannot be maintained prior to a conscious or unconscious initiation of the deceleration effort (Nikolaidis et al. 2018).

Recent advancements in sprint assessment technology, such as motorized linear position encoders, such as the 1080Sprint (1080 Sprint; 1080 Motion, Sweden), offer the potential for alternate analysis methods to assess deceleration performance. Specifically, the availability of a higher sampling rate (333 Hz) (Røkke 2018) and instantaneous velocity‐time data from this equipment enables new methods of deceleration assessment to be created, namely, applying a deceleration threshold, as suggested by D. J. Harper et al. (2023). It is hypothesized that this method may enhance the reliability of the key deceleration metrics by accounting for the potential effects of the cognitive preparation associated with pre‐planned efforts, as well as the possibility that the athlete may prematurely achieve peak velocity prior to actively initiating the deceleration effort. That is, once the athlete achieves their instantaneous peak velocity, they may decline to a slightly slower velocity that does not yet indicate that the deceleration effort has been initiated. Therefore, to assess the viability of the novel deceleration threshold method, the purpose of this study was to compare the intra‐ and inter‐day reliability of this method against the pre‐existing methods. Specifically, these methods are differentiated by how the start point of the deceleration effort is set and include: (1) from the end of the 30 m sprint distance (set distance method); (2) the distance where the instantaneous peak velocity occurred (peak velocity method); and (3) the distance where the acceleration was first ≤ −1.5 m · s^−2^ (deceleration threshold method).

## 2 Materials and Methods

### 2.1 Study Design

To calculate and compare the intra‐ and inter‐day reliability of the three deceleration analysis methods, a repeated‐measures design was employed. Subjects initially attended a familiarization session 1 week prior to the commencement of the study, where they performed at least five trials of the sprint deceleration assessment protocol and had their anthropometric measurements (height and body mass) assessed. Following this, two testing days were completed 1 week apart, with a standardized warm‐up undertaken that consisted of sub‐maximal aerobic exercise, dynamic stretches, and incremental intensity acceleration and deceleration efforts performed at the start of each session. All testing was performed on an undercover concrete surface. Furthermore, subjects were required to wear the same footwear for each testing day to ensure consistency in the shoe‐surface interface between days and restrict themselves from any physical training in the 24 h prior to testing.

### 2.2 Subjects

Twenty subjects (female: *n* = 10, 23.1 ± 4.7 years, 164.3 ± 4.1 cm, 68.7 ± 10.1 kg; and male: *n* = 10, 23.3 ± 3.7 years, 180.6 ± 10.8 cm, and 77.5 ± 9.7 kg) from multiple running‐based field and court team sports (rugby league, rugby union, football, netball, Australian football, touch football, field hockey, and American football) volunteered to participate in this study. To be eligible for inclusion, all subjects were required to be aged between 18 and 35 years old, currently competing at a regional level in their sport, and free of any injury or illness that may have been worsened by their involvement in this study. Prior to providing written informed consent, all subjects were informed of the purpose and requirements of the research and screened for medical contraindications. Ethical approval was obtained through the University of Newcastle Human Ethics Committee (H‐2023‐0013).

### 2.3 Procedures

#### 2.3.1 Sprint Deceleration Assessment

**FIGURE 1:**
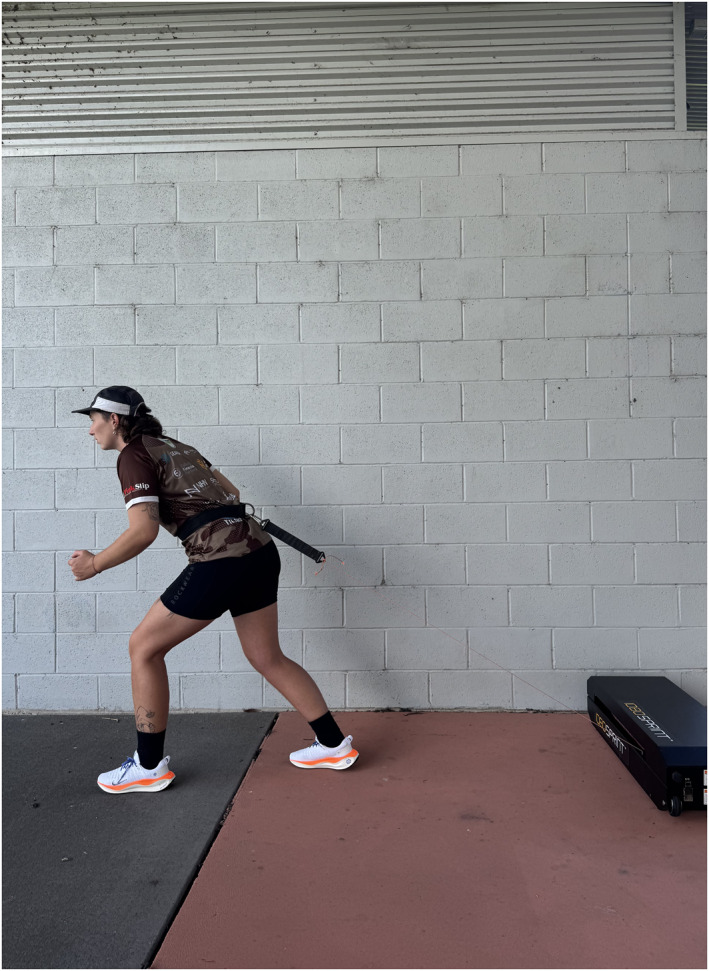
Demonstration of the start position with the 1080Sprint. The participant is positioned approximately 1 m from the device, in a two‐point start position, with the purpose‐built strap attached around their waist and adjusted to fit.

To assess maximal sprint deceleration performance, subjects completed three maximal effort trials of the assessment, separated by at least 3 min of passive rest, on each testing day. For each trial, subjects were required to perform a 30‐m maximal effort sprint to a set of markers, followed by a rapid deceleration to a complete stop. The verbal instructions provided were to “sprint all the way through the markers, before stopping as quickly as possible.” The sprint distance of 30 m was selected as it has been demonstrated to allow athletes to achieve a true sprint peak velocity, and therefore provides a more representative indicator of maximal deceleration capability (Rumpf et al. 2016). Subjects were tethered to a motorized linear encoder device (1080 Sprint; 1080 Motion, Sweden) with a purpose‐built strap attached around their waist, and instructed into a position as displayed in Figure 1 to ensure the cable was taught at the start of the trial. Specifically, this equipment is a cable‐driven resistance system that is connected to a motorized drum, containing a linear position encoder that continuously tracks time and position data up to a length of 90 m (Røkke 2018). From this data, the athletes' instantaneous velocity and acceleration are calculated (Røkke 2018). During all trials the device was set to the “isotonic” mode with 1 kg of resistance to minimize any slack in the cable, which has previously been determined as valid and reliable for assessing maximal sprint performance (Rakovic et al. 2022). The device was interfaced with a portable tablet (Surface Go 2, Microsoft, Remond, USA) through a Bluetooth connection, with all kinetic sprint data uploaded through a cloud‐based system to the proprietary software (1080 Motion Webapp, 1080 Motion, Sweden).

Subjects started each trial with their front foot on a line that was 30 m away from the markers and initiated the trial once the lead researcher (MW) had indicated that they could commence. The recording for each trial commenced when the device detected a change in cable position of > 0.2 m · s^1^ and finished automatically once the subject reached a stationary position. All trials were extracted as comma‐separated value files from the proprietary online server (https://webapp.1080motion.com/) for the raw position‐time data, that was sampled at 333 Hz. To analyze the data, a customized code (R Studio, v2024.04.1 + 748, Boston, Massachusetts) was created for this study, whereby the raw position‐time data were initially filtered using a 200 Hz lowpass‐filter (fourth‐order, zero lag Butterworth Filter), as outlined by Sugisaki et al. (2024) to smooth the data and eliminate any potential spikes. From this filtered data, the instantaneous velocity (Equation 1) and acceleration (Equation 2) were calculated using the following equations:

(1) $$\text{Velocity}\;\left(m\cdot s^{-1}\right)=\frac{d_{f}-d_{i}}{t_{f}-t_{i}}$$

(2) $$\text{Acceleration}\;\left(m\cdot s^{-2}\right)=\frac{v_{f}-v_{i}}{t_{f}-t_{i}}$$

where *d* is position, *v* is velocity, *t* is time, *f* is the final velocity or time, and *i* is the initial velocity or time.

To analyze deceleration performance, the start point of the deceleration effort was determined for the three different methods as follows: (1) from the end of the 30‐m sprint distance (set distance method); (2) the distance where the instantaneous peak velocity occurred (peak velocity method); and (3) the distance where the acceleration data were first ≤ −1.5 m  s^−2^ (deceleration threshold method). The end of the deceleration effort for all methods was recognized as the point where the instantaneous velocity was equal to 0 m · s^−1^. To determine the specific value selected for the deceleration threshold method, pilot data were analyzed using various deceleration thresholds (−1.0, −1.5, and −2.0 m · s^−2^) that were based on those used in team‐sport environments to detect deceleration efforts. Decidedly, −1.5 m s^−2^ was selected as it resulted in the best reliability and when analyzing the force trace it was the point at which the research team was confident represented an intentional deceleration and not an inability to maintain an instantaneous peak velocity.

**FIGURE 2:**
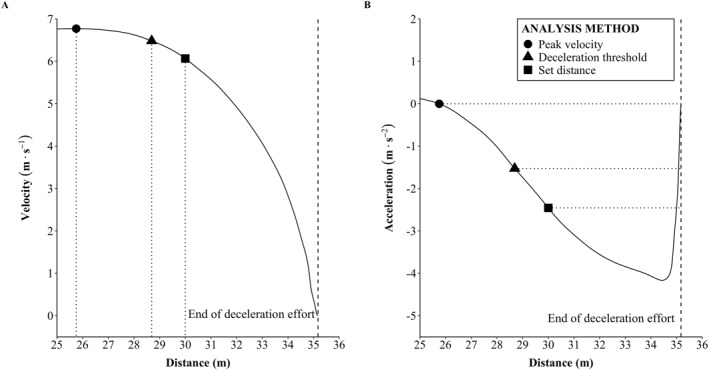
Deceleration effort data obtained from the 1080Sprint from the point of 25 m and onward. (A) The velocity—position curve and (B) the acceleration—position curve. On each figure, the deceleration start point is identified by 30 m (set distance; square), deceleration threshold of −1.5 m · s^−2^ (triangle) and the point of peak velocity (circle). DTS from each start point is identified by the horizontal “dotted” line, and the end position of the deceleration effort (as defined when the subject reached a stationary position) is identified by the vertical “dashed” line.

For each trial and method, the peak sprint velocity was recorded, and the difference between the time and position values at the start and end points of the deceleration effort used to calculate the TTS and DTS, respectively (Figure 2). The best trial for each analysis method, was determined as that with the highest DEC_ave_ value, as suggested by D. J. Harper et al. (2023). DEC_ave_ was calculated by averaging all instantaneous deceleration (negative acceleration) values from the onset of the deceleration effort to point at which the subject comes to a complete stop. Additionally, the velocity decrement from peak velocity to the instantaneous velocity at the start point of the deceleration effort were calculated as a percentage for descriptive purposes.

### 2.4 Statistical Analysis

To determine the intra‐ and inter‐day reliability for the key deceleration metrics of each analysis method, intraclass correlation coefficients (ICC) using two‐way fixed effect models were calculated. The *SimplyAgree* package in R Studio (R Studio, v2024.04.1 + 748, Boston, Massachusetts) was used, with separate analyses conducted to compare the effect of using a different number of trials on the ICC calculations to optimize inter‐day reliability. For intra‐day reliability, all trials from the first testing day were used in the calculation. Moreover, for inter‐day reliability, the best trial from each testing day was determined with the ICC3 (consistency) value, and the average of the best two and average of all three trials were reported using the ICC2k (average agreement) value. Confidence intervals (CI) were calculated for the ICC values and set at 95% (95% CI). Based on the lower to upper bounds of these 95% CI, the reliability values were interpreted with the following criteria; “poor” (< 0.5), “moderate” (0.5–0.74), “good” (0.75–0.9), or “excellent” (> 0.9) (Koo and Li 2016). Furthermore, the coefficient of variation (CV) was calculated to represent the dispersion from the mean for each variable and presented as a percentage (CV%). The CV% was interpreted as “good” (CV < 5%), “moderate” (CV = 5%–10%), or “poor” (CV > 10%) (Duthie, Pyne, and Hooper 2003). Finally, to determine the variability within a subject and the smallest meaningful difference between two testing time points, the standard error of measurement (SEM; $\text{total}\;\text{SD}\sqrt{1-\text{ICC}}$) and minimal detectable change (MDC; $1.96\times \sqrt{2}\times \text{SEM}$) were calculated (Stratford et al. 1998; Weir 2005).

## 3 Results

The mean and standard deviation (SD) of the key deceleration metrics for each analysis method, using the average of the best two trials from each testing day are reported in Table 1. For the intra‐day reliability of the key metrics of TTS, DTS, and DEC_ave_ across all analysis methods, only DTS with the deceleration threshold method was deemed to have acceptable intra‐day reliability (ICC = 0.89, CV% = 6.8, SEM = 0.40 m). Conversely, intra‐day reliability for DTS using the set distance (ICC = 0.87, CV% = 14.0) and peak velocity methods (ICC = 0.40, CV% = 11.3) were considered poor to moderate. For all analysis methods the intra‐day reliability for TTS (ICC = 0.23–0.30, CV% = 14.2–22.1) and DEC_ave_ (ICC = 0.24–0.61, CV% = 12.7–21.0) were considered poor to moderate.

**TABLE 1:** Descriptive statistics (average ± SD) for measures of deceleration performance for the set distance method, deceleration threshold method, and peak velocity method using the best two trials from each testing day.

Analysis method	Testing day	Peak velocity (m · s^−^^1^)	Distance at deceleration start (m)	Velocity at deceleration start (m · s^−1^)	Velocity decrement from peak to deceleration start (%)	TTS (s)	DTS (m)	DEC_ave_ (m · s^−^^2^)	Instantaneous acceleration at deceleration start (m · s^−2^)
Set distance	1	7.18 ± 0.67	30.01 ± 0.01	5.38 ± 0.92	−25.1 ± 10.1	1.30 ± 0.31	2.97 ± 1.09	4.28 ± 0.86	−4.5 ± 1.5
	2	7.19 ± 0.68	30.01 ± 0.01	5.40 ± 1.15	−25.1 ± 13.5	1.38 ± 0.36	3.19 ± 1.44	4.04 ± 0.89	−4.3 ± 1.7
Deceleration threshold	1	7.17 ± 0.68	27.15 ± 0.92	6.88 ± 0.72	−4.2 ± 1.4	1.77 ± 0.30	5.82 ± 1.19	3.98 ± 0.71	−1.5 ± 0.0
	2	7.19 ± 0.68	27.24 ± 1.33	6.91 ± 0.73	−4.0 ± 1.8	1.83 ± 0.38	5.98 ± 1.34	3.91 ± 0.82	−1.5 ± 0.0
Peak velocity	1	7.18 ± 0.67	23.39 ± 1.44	7.18 ± 0.67	—	2.33 ± 0.30	9.62 ± 1.15	3.15 ± 0.59	0.0 ± 0.0
	2	7.22 ± 0.64	23.87 ± 1.98	7.22 ± 0.64	—	2.34 ± 0.46	9.36 ± 1.98	3.23 ± 0.79	0.0 ± 0.0

*Note:* Velocity decrement from peak to deceleration start (%) is not an applicable measure for the peak velocity method, as the start of the deceleration effort is the point where peak velocity occurs.

Abbreviations: DEC_ave_ = average of all instantaneous deceleration values, DTS = distance to stop, TTS = time to stop.

**FIGURE 3:**
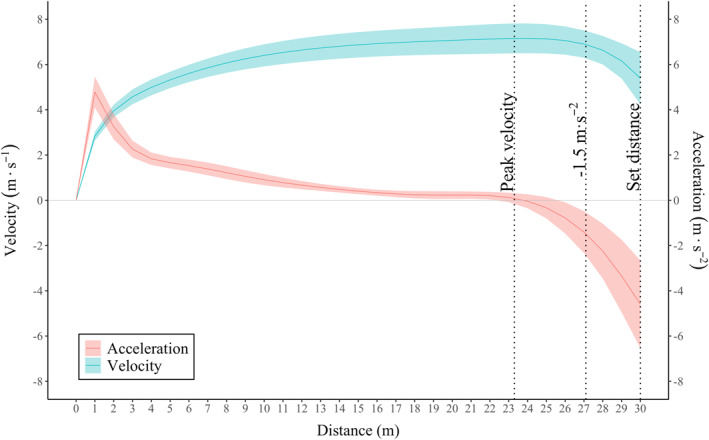
Average ± standard deviation velocity and acceleration for every meter taken across the best trials from each day, with the average deceleration start position for each assessment method.

Across all trials, the mean velocity decrement from peak velocity to the instantaneous velocity at the start point of the deceleration effort was −26.2 ± 12.9% (set distance method), −4.4 ± 1.8% (deceleration threshold method), and 0.0 ± 0.0% (peak velocity method). This corresponded with a mean start distance and instantaneous acceleration of; 30.0 ± 0.0 m and −4.4 ± 1.6 m · s^2^, 27.1 ± 1.2 m, and −1.5 ± 0.0 m · s^2^, and 23.3 ± 1.9 m and 0.0 ± 0.0 m · s^2^, respectively (Figure 3). Importantly, the inter‐day reliability for the instantaneous velocity at the start point of the deceleration effort was considered excellent for the deceleration threshold and peak velocity methods, but poor for the set distance method.

The inter‐day reliability measures for each analysis method using the best trials, average of best two trials, and average of all three trials are presented separately in Tables 2, 3, 4. For DTS, TTS, and DEC_ave_, excellent reliability was only identified for all key outcome metrics when analyzing the average of the best two trials with the deceleration threshold method. Further, excellent reliability was calculated for DTS when using the average of the best two trials for the set distance method, as well as the average of all three trials for both the deceleration threshold and set distance methods.

**TABLE 2:** Inter‐day reliability and sensitivity of linear encoder derived variables collected from the best trial in each day.

Method	Variable	CV%	SEM	MDC	ICC (95% CI)
Set distance	Peak velocity (m · s^−1^)	1.8	0.13	0.37	0.96 (0.92–0.98)
	Deceleration start velocity (m · s^−1^)	12.7	0.69	1.92	0.55 (0.23–0.76)
	Velocity decrement (%)	38.8	9.37	25.97	0.39 (0.02–0.66)
	Deceleration start position (m)	0.0	0.00	0.01	0.00 (−0.37–0.37)
	TTS (s)	16.2	0.21	0.57	0.56 (0.24–0.77)
	DTS (m)	24.5	0.77	2.13	0.65 (0.37–0.82)
	DEC_ave_ (m · s^−2^)	12.2	0.54	1.50	0.62 (0.33–0.81)
Deceleration threshold	Peak velocity (m · s^−1^)	1.8	0.13	0.37	0.96 (0.92–0.98)
	Deceleration start velocity (m · s^−1^)	2.3	0.16	0.44	0.95 (0.89–0.98)
	Velocity decrement (%)	26.6	1.07	2.97	0.57 (0.26–0.78)
	Deceleration start position (m)	3.1	0.83	2.31	0.53 (0.20–0.75)
	TTS (s)	11.8	0.20	0.55	0.62 (0.33–0.81)
	DTS (m)	7.8	0.45	1.24	0.87 (0.74–0.94)
	DEC_ave_ (m · s^−2^)	12.0	0.50	1.39	0.61 (0.31–0.80)
Peak velocity	Peak velocity (m · s^−1^)	1.8	0.13	0.36	0.96 (0.92–0.98)
	Deceleration start velocity (m · s^−1^)	1.8	0.13	0.36	0.96 (0.92–0.98)
	Velocity decrement (%)	—	—	—	—
	Deceleration start position (m)	5.5	1.31	3.63	0.08 (−0.30–0.43)
	TTS (s)	12.1	0.27	0.74	0.38 (0.02–0.66)
	DTS (m)	14.6	1.33	3.69	0.19 (−0.19–0.52)
	DEC_ave_ (m · s^−2^)	15.3	0.52	1.43	0.49 (0.15–0.73)

Abbreviations: CV% = coefficient of variation, DEC_ave_ = average of all instantaneous deceleration values, DTS = distance to stop, ICC = intra‐class coefficient, MDC = minimal detectable change, SEM = standard error of measurement, TTS = time to stop.

**TABLE 3:** Inter‐day reliability and sensitivity of linear encoder derived variables collected from the average of the best two trials in each day.

Method	Variable	CV%	SEM	MDC	ICC (95% CI)
Set distance	Peak velocity (m · s^−1^)	1.3	0.09	0.26	0.99 (0.98–0.99)
	Deceleration start velocity (m · s^−1^)	7.4	0.40	1.11	0.92 (0.86–0.95)
	Velocity decrement (%)	22.0	5.53	15.33	0.88 (0.79–0.93)
	Deceleration start position (m)	0.0	0.01	0.01	0.13 (−0.48–0.49)
	TTS (s)	10.5	0.14	0.39	0.90 (0.83–0.94)
	DTS (m)	13.6	0.42	1.16	0.94 (0.90–0.97)
	DEC_ave_ (m · s^−2^)	8.1	0.34	0.93	0.91 (0.85–0.95)
Deceleration threshold	Peak velocity (m · s^−1^)	1.3	0.09	0.26	0.99 (0.98–0.99)
	Deceleration start velocity (m · s^−1^)	1.8	0.12	0.33	0.99 (0.98–0.99)
	Velocity decrement (%)	24.4	1.00	2.77	0.76 (0.60–0.86)
	Deceleration start position (m)	1.6	0.43	1.19	0.92 (0.87–0.96)
	TTS (s)	6.4	0.12	0.32	0.93 (0.89–0.96)
	DTS (m)	5.3	0.31	0.86	0.97 (0.95–0.98)
	DEC_ave_ (m · s^−2^)	7.1	0.28	0.78	0.92 (0.86–0.95)
Peak velocity	Peak velocity (m · s^−1^)	1.2	0.08	0.23	0.99 (0.99–1.00)
	Deceleration start velocity (m · s^−1^)	1.2	0.08	0.23	0.99 (0.99–1.00)
	Velocity decrement (%)	—	—	—	—
	Deceleration start position (m)	5.1	1.21	3.35	0.67 (0.44–0.81)
	TTS (s)	7.8	0.18	0.50	0.86 (0.76–0.92)
	DTS (m)	11.7	1.11	3.08	0.67 (0.44–0.81)
	DEC_ave_ (m · s^−2^)	7.0	0.23	0.62	0.94 (0.90–0.97)

Abbreviations: CV% = coefficient of variation, DEC_ave_ = average of all instantaneous deceleration values, DTS = distance to stop, ICC = intra‐class coefficient, MDC = minimal detectable change, SEM = standard error of measurement, TTS = time to stop.

**TABLE 4:** Inter‐day reliability and sensitivity of linear encoder derived variables collected from the average of the 3 trials in each day.

Method	Variable	CV%	SEM	MDC	ICC (95% CI)
Set distance	Peak velocity (m · s^−1^)	1.5	0.10	0.28	0.99 (0.99–0.99)
	Deceleration start velocity (m · s^−1^)	8.5	0.45	1.26	0.94 (0.90–0.96)
	Velocity decrement (%)	23.6	6.10	16.91	0.92 (0.87–0.95)
	Deceleration start position (m)	0.0	0.01	0.02	0.29 (−0.11–0.56)
	TTS (s)	19.5	0.28	0.78	0.72 (0.57–0.83)
	DTS (m)	15.3	0.48	1.32	0.95 (0.93–0.97)
	DEC_ave_ (m · s^−2^)	18.8	0.73	2.02	0.65 (0.46–0.79)
Deceleration threshold	Peak velocity (m · s^−1^)	1.5	0.10	0.28	0.99 (0.99–0.99)
	Deceleration start velocity (m · s^−1^)	2.0	0.13	0.37	0.99 (0.98–0.99)
	Velocity decrement (%)	22.3	0.91	2.53	0.85 (0.77–0.91)
	Deceleration start position (m)	2.1	0.56	1.55	0.91 (0.87–0.95)
	TTS (s)	16.5	0.32	0.87	0.68 (0.51–0.80)
	DTS (m)	7.0	0.42	1.16	0.96 (0.94–0.98)
	DEC_ave_ (m · s^−2^)	15.3	0.57	1.58	0.71 (0.54–0.82)
Peak velocity	Peak velocity (m · s^−1^)	1.5	0.10	0.29	0.99 (0.99–0.99)
	Deceleration start velocity (m · s^−1^)	1.5	0.10	0.29	0.99 (0.99–0.99)
	Velocity decrement (%)	—	—	—	—
	Deceleration start position (m)	5.7	1.33	3.69	0.81 (0.71–0.88)
	TTS (s)	14.9	0.37	1.01	0.64 (0.45–0.78)
	DTS (m)	12.8	1.25	3.46	0.77 (0.64–0.85)
	DEC_ave_ (m · s^−2^)	14.5	0.44	1.22	0.82 (0.72–0.89)

Abbreviations: CV% = coefficient of variation, DEC_ave_ = average of all instantaneous deceleration values, DTS = distance to stop, ICC = intra‐class coefficient, MDC = minimal detectable change, SEM = standard error of measurement, TTS = time to stop.

## 4 Discussion

The primary aim of this study was to compare the intra‐ and inter‐day reliability of three different analysis methods for a 30‐m sprint deceleration assessment. Specifically, these included the set distance method, the peak velocity method, and the novel deceleration threshold method. For the key deceleration metrics of DTS, TTS, and DEC_ave_, intra‐day reliability was only acceptable for DTS using the deceleration threshold method (ICC = 0.89, CV% = 6.8). Further, inter‐day reliability was excellent and the MDC for TTS and DTS were smallest for the deceleration threshold when using the average of the best two trials from each day, further supporting this method as valid, reliable, and sensitive for linear deceleration assessments. Comparatively, when considering the descriptive statistics, it appears that there is greater variability (higher CV%) for the set distance method as the subjects had already initiated the deceleration effort well before the 30‐m mark, as evidenced by the mean instantaneous acceleration of −4.4 ± 1.6 m · s^2^ at this point. Moreover, the limited reliability for the peak velocity method is likely explained by the moderate reliability for the start point of the deceleration effort. Specifically, some subjects appeared to have achieved their peak velocity early in the 30‐m sprint prior to reaccelerating, albeit to a lower instantaneous velocity, prior to initiating the deceleration effort. Together, this data encourages the use of the deceleration threshold method using the best trial for DTS, to optimize sprint deceleration assessment reliability.

The results of this study align with those of D. J. Harper et al. (2023), in which the subjects frequently initiated the deceleration effort prior to the end of the set sprint distance. For the set distance method, the mean decrement from the peak velocity to the instantaneous velocity at the start of the deceleration effort in this study was −25.1 ± 11.9%. Additionally, at the 30‐m mark, the mean instantaneous deceleration was −4.4 ± 1.6 m · s^2^ (range: −1.1 to −9.5 m · s^2^), indicating that the deceleration effort was initiated well before the instructed start position. Although D. J. Harper et al. (2023) suggested the use of the peak velocity method to overcome these concerns with the set distance method, the present data highlights limitations with this alternate method, given that on average the peak velocity, and hence start point of the deceleration effort was recorded at 23.3 ± 1.9 m. Further analysis of this method revealed that numerous subjects experienced a small magnitude of deceleration, prior to a re‐acceleration without surpassing the peak velocity already achieved. Conversely, when using the deceleration threshold method, the start position of the deceleration effort was determined at 27.1 ± 1.2 m, which appears more logical when considering the likelihood that the subjects may cognitively prepare for the rapid deceleration. Furthermore, the velocity decrement using the deceleration threshold method was only −4.1 ± 1.6%, suggesting that this method provides a more accurate representation of the subjects' maximal deceleration capability. Therefore, it is likely that the reduced reliability for the set distance and peak velocity methods is due to an inaccurate determination of the start of the deceleration effort. Specifically, for the set distance method the data indicates that most subjects had initiated the deceleration effort prior to the 30‐m mark, whereas for the peak velocity method it does not consistently indicate that active deceleration has been initiated immediately following the point at which the peak velocity occurs.

Accurate determination of the start point of a deceleration effort is crucial for precise profiling of athletes during a pre‐planned deceleration assessment. As the key deceleration metrics of interest to practitioners (DTS, TTS, and DEC_ave_) are distance and time dependent, it is evident that inaccuracy in the determination of this start point will likely lead to reduced reliability of these metrics (D. J. Harper et al. 2023). In this study, the deceleration threshold was the only analysis method identified as having acceptable intra‐day reliability for DTS (ICC = 0.89, CV% = 6.8), and inter‐day reliability for all key deceleration metrics when using the average of the best two trials. It is suggested that this is because the use of a set deceleration threshold provided a more accurate determination of the start point than both the set distance and peak velocity methods, as it accounted for potential unconscious cognitive preparation of the deceleration effort. The set threshold of deceleration (negative acceleration) used for this analysis was < −1.5 m · s^2^, which was based upon the threshold employed in GNSS analysis of field‐based team sport athletes (Blair, Body, and Croft 2017). Although future research may explore other thresholds to potentially improve either the determination of the start of the deceleration effort or the suitability for specific sports, this study provides initial data that highlights the value of this analysis method for enhancing the reliability and ability to detect a meaningful change in sprint deceleration performance.

Utilizing a pre‐planned linear sprint acceleration to deceleration tasks allows for an independent assessment of maximal deceleration capability, regardless of any COD‐constrained technique (D. J. Harper et al. 2023). The key deceleration metrics of interest have been identified as DTS, TTS, and DEC_ave_, with Ashton and Jones (2019) reporting that DTS of an entire deceleration effort has a greater level of reliability compared to analyzing deceleration distances to certain velocity decrements. Supporting this suggestion, the present data highlights that DTS had the best inter‐day reliability across all trials when using the deceleration threshold method (ICC = 0.87 to 0.97, CV% = 5.3–7.8), further reinforcing its suitability as the key metric for profiling and monitoring maximal sprint deceleration capability. However, it is important to note that when using this analysis method, all key metrics were identified as displaying excellent reliability (TTS: ICC = 0.93, CV% = 6.4; DEC_ave_: ICC = 0.92, CV% = 7.1). Therefore, these results suggest that practitioners may utilize any of the key metrics to determine the effectiveness of a training intervention due to the acceptable reliability but should consider the ability to detect a meaningful change in performance when selecting the metric of importance. However, the reported data are a representation of this specific cohort and their respective athletic abilities and sporting background, so it is recommended that future research aims to determine whether this analysis method and metric are most appropriate for optimizing deceleration assessment reliability in other cohorts.

Anecdotally, an important consideration for enhancing reliability with performance assessments is whether to use the best trial or the average of multiple trials. D. J. Harper et al. (2023) previously reported that the best trial for deceleration assessments should be those with the highest DEC_ave_, as this factors in both the distance and time components of performance. However, to the best of our knowledge no previous research has compared the inter‐day reliability of the key deceleration metrics when using the best trial or average of multiple trials. In the present study, using the average of the best two trials with the deceleration method displayed superior reliability to all other analysis methods and use of the best or all three trials, with substantially lower MDCs was substantially lower. Specifically, for the metrics of TTS, DTS, and DEC_ave_ with the deceleration threshold methods, the MDC was 0.32 s, 0.86 m, and 0.78 m · s^−2^ (average of best two), compared to 0.55 s, 1.24 m, and 1.39 m · s^−2^ for the best trial, and 0.87 s, 1.16 m, 1.58 m · s^−2^ for the average of all three. Together, this suggests that the deceleration threshold method using the average of the best two trials for all key deceleration metrics appears to optimize reliability and the ability to detect a meaningful change in performance. However, further research is required with a variety of other cohorts to confirm this, as these findings may have been associated with the experience and athletic ability of the present subjects.

Although this study provides valuable insights into linear deceleration assessments, several limitations should be considered when applying the findings. The specific threshold of ≤ −1.5 m · s^−2^ was selected following pilot testing on a relatively small sample size from a broad range of multi‐directional team sports. Future research is warranted to investigate the suitability for this threshold for assessing a group of athletes from a specific sport. Additionally, different thresholds may influence the reliability of this assessment, especially for athletes with a faster or slower peak velocity. Finally, this study employed a pre‐planned deceleration and as such the application of this method of analysis in reactive assessments may not be appropriate. Therefore, future research into the applicability of this method with reactive assessments is warranted.

## 5 Conclusion

This study compared three different analysis methods for assessing sprint deceleration performance to determine the optimal method to enhance intra‐ and inter‐day reliability. The data from this study may be used by practitioners to select sprint deceleration analysis methods and metrics to enhance reliability and the ability to detect a meaningful change in performance. Importantly, the novel deceleration threshold method demonstrated the best intra‐day reliability and inter‐day reliability when using the average of the best two trials for all key deceleration metrics. The descriptive data of this study suggests that most subjects had initiated the deceleration effort prior to the 30‐m mark and did not consistently initiate active deceleration immediately following the point at which their peak velocity was recorded. This likely explains the lower reliability for the set distance and peak velocity methods. Although future research should determine whether similar findings are observed in other athletic cohorts, implementing the data from this study may enable practitioners to determine the effectiveness of training interventions and guide rehabilitation programs aimed at enhancing athlete performance and health.

## Ethics Statement

Ethical approval was granted by the University of Newcastle Human Ethics Committee (H‐2023‐0013), and all subjects provided informed consent prior to the commencement of the study.

## Conflicts of Interest

The authors declare no conflicts of interest.

## Data Availability

The data that supports the findings of this study are available from the corresponding author, MW, upon reasonable request.
